# The Impact on the Stress-Associated Autonomic Response of Physiotherapy Students Receiving Interferential Current in an Electrotherapy Training Session

**DOI:** 10.3390/ijerph192013348

**Published:** 2022-10-16

**Authors:** Luis Espejo-Antúnez, Carlos Fernández-Morales, Sergio Hernández-Sánchez, María de los Ángeles Cardero-Durán, José Vicente Toledo-Marhuenda, Manuel Albornoz-Cabello

**Affiliations:** 1 Department of Medical-Surgical Therapeutics, Faculty of Medicine and Health Sciences, University of Extremadura, 06006 Badajoz, Spain; 2 Department of Pathology and Surgery (Area of Physiotherapy), Faculty of Medicine, Miguel Hernández University, 03550 Alicante, Spain; 3 Department of Physical Therapy, Faculty of Nursing, Physical Therapy and Podiatry, University of Seville, 41009 Seville, Spain

**Keywords:** students, physiotherapy, interferential current, heart-rate variability, autonomic nervous system, physiological stress

## Abstract

Electrical currents are didactic contents widely applied in the training of physiotherapy students, but the treatment is considered a stressful situation for both the patient who receives it and the student who applies it. The aim of this study was to evaluate the stress-associated autonomic response of physiotherapy students receiving interferential current by measuring and analysing heart rate variability. An observational case–control study was conducted. Ninety healthy male volunteers, all physiotherapy degree students, were enrolled while attending laboratory practice during the 2020–2021 academic year. Participants were randomly allocated to a sham electrotherapy group (44 subjects), in which heart rate variability was recorded for 10 min, both at rest and during the application of sham technique on the lower back (10 min), and an electrotherapy group (46 subjects), applying the same procedure with the electrical current flowing. Outcome measures included baseline (seated position) and postintervention (prone position) time domain parameter, diameters of the Poincaré plot 1 and 2, stress score, and sympathetic/parasympathetic ratio. The sham electrotherapy group exhibited significant increases in time domain parameter (*p* = 0.027) and diameters of the Poincaré plot 1 (*p* = 0.032), with a small effect size (d ≤ 0.5). The electrotherapy group exhibited significant increases in time domain parameter and diameters of the Poincaré plot 1 and 2 (*p* < 0.001) and decreases in the stress score and sympathetic/parasympathetic ratio (*p* < 0.001), with a large effect size (d > 0.8) other than for the time domain parameter (d = 0.42), indicating increased parasympathetic and decreased sympathetic activity. After interventions, there were significant differences between groups in diameters of the Poincaré plot 2 (*p* < 0.001), stress score (*p* = 0.01) and sympathetic/parasympathetic ratio (*p* = 0.003), with moderate effect size (d > 0.5). The application of the interferential current technique produces stress-associated autonomic response characterized by greater parasympathetic activity and decreased sympathetic activity. Further studies are needed to determine possible adverse effects.

## 1. Introduction

Stress comprises a state of emotional and physical tension caused by demands that exceed the usual response capacity. It produces a homeostatic disturbance that leads to physical and psychological changes [1]. Consequently, mechanisms are produced to be re-established by the organism [2]. The autonomic nervous system (ANS) is responsible for regulating the stress response. There is an increase in the sympathetic activity with consequent physiological changes (increased heart rate, respiratory rate, blood pressure, etc.). In order to restore the homeostasis, an increase in the activity of the parasympathetic nervous system takes place once the stressors have disappeared [3].

From a cognitive standpoint, a stressed individual finds it difficult to concentrate and maintain focus; struggles with decision-making; and exhibits irritability, anxiety, and worry [4]. In this sense, the learning process of students in health sciences has been related to habituating to stressful situations. Previous studies in different academic settings have measured the psychophysiological demands on students and learning outcomes [1,5,6,7].

Morales et al. [5] observed inhibition of the parasympathetic system and increased sympathetic activity in residents physicians with excessive time dedicated to training (24 h without sleep). Nakayama et al. [6] showed significant increases in sympathetic activity during a simulated clinical practice in nursing students. On the other hand, Stein [8] observed a decrease in parasympathetic activity when evaluating simulated practice in emergency medicine students.

Among other health sciences degrees, physiotherapy stands out in the application of simulation scenarios in laboratory practice [9]. Beltrán-Velasco et al. [10] reported that the stress response influences negatively, affecting memory, decision-making, and learning. These physiological responses derived from stressful situations have been observed in specific clinical learning contexts.

Electrotherapy is commonly used in physiotherapy clinical practice, and it is also widely used in training among students. This academic experience is stressful, but it allows students to acquire essential professional skills and competences, as the repeated exposure facilitates the habituation process [11]. These experiences try to offer a real but controllable environment in which the students face a clinical situation as they will in real future situations without risk for the patient [8].

The interferential current (IFC) technique is frequently used as a therapeutic medium-frequency electrical transcutaneous stimulation procedure (4 kHz) [12,13]. Physiological changes caused include an increase in blood flow, effects on the peripheral circulation, and parameters related to the autonomic nervous system such as heart rate variability (HRV) [14,15,16,17]. 

HRV is an useful tool for analysing the stress-associated autonomic response between sympathetic and parasympathetic activity in clinical and research studies [18]. As a result of computerized ECG acquisition systems and software, HRV provides an accessible and easy-to-use set of parameters for evaluating the ANS balance [19]. There is currently a growing interest in monitoring psychophysiological markers such as HRV in the university setting due to the effects observed when activities requiring a certain level of cognitive demand are carried out [20]. Souza et al., (2019) [21] indicated the possibility of estimating the state of stress in activities related to this area.

Given the above, the aim of this study was to assess the autonomic response (analysed through HRV) of physiotherapy students receiving IFC technique on the lower back in a training session. 

## 2. Materials and Methods

### 2.1. Study Design

An observational case–control study was conducted to compare parameters related to sympathetic and parasympathetic activity (analyzed through HRV) while applying the IFC technique in healthy physiotherapy students. The experiment was carried out in the practical classroom where the equipment is located and where students are trained in the use of electrophysical agents. The intervention was carried out just before the beginning of school hours (9:00 a.m.) to try to reduce possible stressors derived from cognitive demands related to academic tasks. The study was performed in accordance with the Strengthening the Reporting of Observational Studies in Epidemiology (STROBE) statement [22].

### 2.2. Participants

An initial, potentially eligible sample of 94 physiotherapy students (all healthy, male adult volunteers) was recruited. The inclusion criteria were: (i) male students enrolled in the bachelor’s degree program in physiotherapy or (ii) in the electrotherapy course (iii) who had completed at least five laboratory practices on the subject prior to the study. Exclusion criteria were: (i) students with a score higher than 37.5 points on the Personal Psychological Apprehension Scale (PPAS) [23]; (ii) any uncontrolled neurological or cardiac disorder that could produce variations in the basal respiratory rate; (iii) other disorders secondary to taking any medication or other medical causes that contraindicated electrical stimulation (e.g., epilepsy), or (iv) modifications in regular physical activity routines that lead to ineligibility to participate, as determined by the investigator.

**Figure 1:**
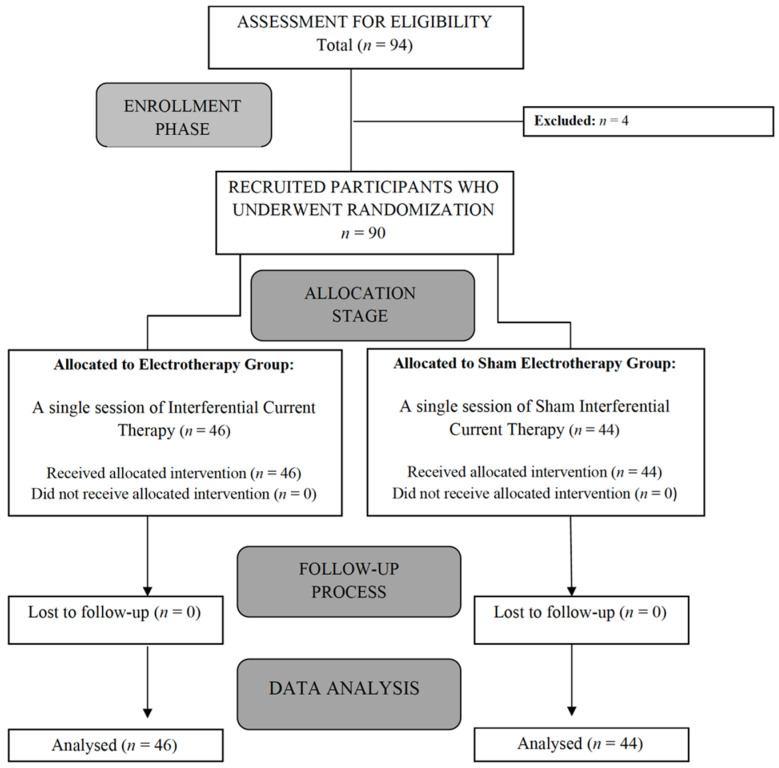
Flow diagram of patient recruitment.

After applying the inclusion/exclusion criteria, the final sample comprised 90 healthy male students aged between 18 and 26 years old (M = 21; SD = 4.67). The recruitment period was from 1 October 2020 to 31 January 2021. Figure 1 provides a flow chart of subject recruitment during the study. 

The local ethics committee (University Hospital Virgen Macarena-Virgen del Rocío) approved the study, which complied with all the principles set forth in the Declaration of Helsinki. All subjects signed an informed consent to participate in this study.

### 2.3. Randomization

An external website (http://www.randomization.com (accessed on 1 September 2020)) was used to complete the group allocation. Participants were randomly (using block randomization, 1:1) allocated into 1 of the 2 groups created: electrotherapy group (EG) and sham electrotherapy group (SEG). The randomization was performed by an external assistant. The participants had to pick a number out of opaque envelopes. Two researchers carried out the experimental phase of the study. One of them, a blinded researcher, collected the measurements, and the other one conducted the intervention in both groups.

### 2.4. Procedures

To monitor the intervention, a self-report was recorded regarding sensations and previous experiences of the sample subjects. For this, all participants completed the PPAS and the Laboratory Environment Questionnaire (LEQ) (Likert type). The PPAS was used to measure self-positioning and the individual’s degree of apprehension regarding the application of electrophysical agents [23]. The LEQ is a validated scale used to analyse the classroom environment in laboratory practice [24]. In addition, as the respiratory rate is considered a vital sign that varies in undergraduate health science students during clinical activities [25], this variable was monitored by a blinded evaluator.

Subsequently, we analysed each student at two points during the experiment. First, HRV was recorded for 10 min at rest in prone position. Then, HRV was recorded in the EG simultaneously with the application of the IFC technique in the lumbar region for 10 min. In the SEG, HRV was recorded during a sham intervention (without application of electric current) on the same body region and for the same time. The experiment was conducted first thing in the morning, just before the start of the school day (9:00 a.m.).

The intervention protocol using the IFC technique was that proposed in a previous study [26]. A Sonopuls 692^®^ device (Enraf-Nonius BV, Rotterdam, The Netherlands) was used to apply a transregional interferential current protocol. The subjects were placed in prone position, with the lumbar region uncovered. Four self-adhesive electrodes type Pals Platinum©, Axelgaard Manufacturing Co. Ltd., Fallbrook, CA, USA (10 × 12 cm, and 75 cm^2^ surface area) were used and placed at the level of the first and fifth lumbar vertebrae, in a cross pattern, plus an automatic vector, two intersection channels, and a 1/1 rectangular pulse. The parameters used were as follows: (a) 4000 Hz carrier frequency; (b) 65 Hz amplitude modulated frequency; and (c) 95 Hz modulation sweep frequency with a 1:1 oscillation pattern, using a quadripolar technique. The intensity of the current was increased to the patient’s tolerance limit. It was described that he should experience a “pins and needles” sensation, avoiding visible muscle contraction. Subjects allocated to the SEG were not verbally informed about any perception or sensation to be noticed when applying the procedure. To avoid the risk of contamination between participants, the two groups were assessed in separate rooms. None of the participants in the study had received theoretical or practical training about the procedure applied before the intervention.

### 2.5. Outcome Measures of Heart Rate Variability

Interbeat time interval (R-R) variation was used to determine the autonomic modulation using Firstbeat Bodyguard equipment (Firstbeat Technologies, Jyväskylä, Finland). HRV data were recorded for 10 min during each session. Recordings were exported from the devices to the computer via Firstbeat Uploader Software (Firstbeat Technologies) and analysed using Kubios Software (University of Eastern Finland, Kuopio, Finland). To calculate the stress-associated autonomic response, we used the HRV method commonly used for this purpose, which is based on the Poincaré plot [27,28]. This software has demonstrated its validity and is capable of registering nonlinear trends that are often presented in registers of variation in the time interval between beats (R-R) [29]. HRV is considered a validated and accurate tool for the assessment of the sympathetic and parasympathetic nervous system in various conditions [30,31].

In addition, in order to improve the reporting of assessment and intervention results through the use of HRV, a checklist for the use of HRV collection and analysis methodology was followed [32]. The mean heart rate and time domain parameters were measured: rMSSD (the square root of the average of the sum of the differences squared between normal adjacent); pNN50 (percentage of consecutive RR intervals that differ by more than 50 ms from each other); diameters of the Poincaré plot, and SD1 (sensitivity of the short-term variability of the non-linear range of the HRV) and SD2 (long-term variability of the non-linear range of the HRV). SD1 is considered an indicator of parasympathetic activity [33]. SD2 is considered an inverse indicator of sympathetic activity reflecting long-term changes in RRI [28]. The stress score (SS) and the sympathetic–parasympathetic ratio (S/PS) are variables defined by Naranjo-Orellana et al. (2005) [34] as facilitating the interpretation of the Poincaré plot. The SS determines the degree of sympathetic activity in the sinus node. It is calculated by the inverse of SD2 multiplied by 1000. The S/PS ratio determines the level of autonomic balance (ratio of sympathetic to parasympathetic activity). It is calculated by the quotient of SS and SD1.

### 2.6. Statistical Analysis

For each variable, the mean, standard deviation and 95% CI were detailed. The Kolmogorov–Smirnov test was used to assess the normality of the data distribution. Previously, a mixed-model analysis of variance (ANOVA) (2 × 2) was used to analyse the data with one between-group factor (sham electrotherapy group versus electrotherapy group) and one within-group factor (baseline versus intervention). Effect sizes (ES) were calculated using Cohen’s d coefficient. An effect size between ≥0.2 and <0.5 reflects a small difference, between ≥0.5 and <0.8 a moderate difference, and ≥0.8 a large difference [35]. Statistical Package for the Social Sciences (SPSS) v.21 (SPSS Inc., Chicago, IL, USA) software was used to analyse the data and statistical significance was set at *p* < 0.05.

### 2.7. Sample Size Estimation

GPower 3.1. software was used to estimate the sample size. An expected sample size of 94 physiotherapy students was assumed. Assuming a one-tailed hypothesis. an effect size (d) of 0.55, an alpha level of 0.05. and power of 80%, a total sample. of 84 participants was estimated. The sample was inflated by 10% to account for potential dropouts, giving a final target sample size of 90 students.

## 3. Results

Table 1 indicates the descriptive statistics of the demographic and anthropometric variables for the electrotherapy and sham electrotherapy groups, including age, weight, height, and body mass index. Analysis of the PPAS and LEQ scale scores found no significant differences between the sham electrotherapy and electrotherapy groups in any of the demographic variables (*p* > 0.05).

Table 2 shows the baseline post-intervention scores and the mean differences in the between-group and within-group comparisons for HRV parameters and percentage change for each of the variables in both groups. There were no differences between the SEG and the EG in the baseline measurements (*p* > 0.05). However, both groups showed significant differences after interventions. The comparison within the sham electrotherapy group revealed a significant increase in rMSSD (5.97 ± 16.24 ms, *p* = 0.019, d = 0.27) and SD1 (6.92 ± 12.67 ms, *p* = 0.001, d = 0.51). The EG exhibited a significant increase in rMSSD (7.91 ± 13.49 ms, *p* < 0.001, d = 0.42), SD1 (12.99 ± 9.43 ms, *p* < 0.001, d = 0.92) and SD2 (24.71 ± 28.35 ms, *p* < 0.000, d = 0.91), and a significant decrease in SS (2.74 ± 5.78 ms, *p* = 0.002, d = 0.51) and S/PS ratio (0.30 ± 0.40, *p* < 0.000, d = 0.86) after the intervention.

Comparison between groups showed significant differences in SD2 (17.03 [5.30 to 28.71] ms, *p* = 0.005, d = 0.61), SS (−3.85 [−6.66 to −1.20], *p* = 0.005, d = 0.60) and S/PS ratio (−0.20 [−0.34 to −0.07], *p* = 0.003, d = 0.70). 

## 4. Discussion

In the last decade, new teaching and assessment models have been consolidated in higher education such as clinical simulation, objective structured clinical evaluation (OSCE), gamification, and role playing. Most models are aimed at finding out the reported benefits of their use when the student plays an active leadership role in the training [7,10,36]. However, it also seems of interest to know the effects when the student is trained adopting a passive role, which is common in some of the methodologies described above. The main finding of this study was to analyse the stress-associated autonomic response in healthy male physiotherapy students while receiving the IFC technique on the lower back.

These findings are relevant since there is no consensus regarding the autonomic responses shown in students from different health science degrees. The students of the degree in physiotherapy usually undergo practical training in the procedures they will apply as health professionals. Training as students involves the simulated execution and reception of agents, techniques, and methods during their undergraduate training. This fact leads to differences with other studies carried out on university students, as the design of these studies varies when measuring the student’s response to stress by adopting an active role in practical training [10,36,37]. 

Previous studies have observed increased performance in physiotherapy students adopting a patient role in terms of understanding their own learning, improving clinical practice skills, and enabling them to educate patients [38,39]. With the association having been established between autonomic balance and academic performance [20], the results obtained with the reduction of stress levels (characterized by a decrease in sympathetic activity) indicate the possible benefit in academic performance through meaningful experiential learning in physiotherapy students, as indicated by Forbes et al. [39]. In this sense, Mandrusiak et al. [38] observed in third-year physiotherapy students who acted/trained as patients an increase in satisfaction with the learning experience, reducing the stress derived from the transition to clinical environments.

Our results are consistent with those reported by Beltrán-Velasco et al. [40] for psychology students. In both studies, SD2 increased significantly, and these changes can be interpreted as showing a decrease in sympathetic activity. However, our results differed from those reported by Sánchez-Conde et al. [1] in nursing students and Beltrán-Velasco et al. [10] in physiotherapy students. These authors reported that the students showed no reduction in their levels of sympathetic activity and no increase in parasympathetic activity, whereas our electrotherapy group showed significant increases and a moderate effect size in the pNN50 parameter (d = 0.74) and large effect size in SD2 (d = 0.99) and decreases with a moderate effect size in SS (d = 0.67), as well as significant increases with large effect sizes in the SD1 parameter (d = 0.93) and S/PS ratio (d = 0.82). Regarding the between-group comparison, we found significant changes (*p* < 0.05) in the variables related to sympathetic activity with a significant increase in SD2 and significant decreases in SS and S/PS ratio. This fact could be due to two different reasons: (i) the neurophysiological effects produced by the interferential current [17] or (ii) the habituation generated by the practical experience accumulated by the students in receiving electrophysical agents up to the beginning of the study. 

Due to the association established between respiratory rate, tidal volume, and vasovagal parameters [41], the use of protection measures used as a consequence of the health crisis caused by SARS-CoV-2 could be considered factors to take into account. Accordingly, the possible impact of the use of surgical masks on the autonomic nervous system of university students during classes with a similar duration (150 min) to the classes in which this study took place has also been analysed and the results did not indicate any effect associated with the parameters evaluated in the present study [20]. However, more studies are needed to assess the impact of other protection measures, such as the use of personal protective equipment (PPE) or social isolation for periods of confinement, for example.

Faced with the new reality that we are living with around the world, we consider that by controlling the possible changes associated with respiratory rate, the differences observed in sympathetic and parasympathetic activity could be due to: (i) the influence of the interferential current on the autonomic response of the students; and (ii) the role of the student in practice. In the present study, all the participating students played a passive role, acting as a patient instead of as a professional. The lack of variation in the basal respiratory rate observed in our study, unlike others [25], suggests the need for future studies to determine the impact of individual protection measures (surgical masks, FPP2 masks, gloves, uniforms…) implemented in the university environment during classes and in the laboratory, as well as the effect produced by electrophysical agents (e.g., interferential current therapy) on autonomous activity, and the effect of the classroom environment.

In this context, the influence of the IFC seems to differ between studies. While Hee-Kyung et al. [14] and Noble et al. [15] observed an increase in sympathetic activity after applying the IFC technique, our results demonstrated significant changes in both parasympathetic and sympathetic activity. This could be due first to the dosage of the application procedure since the appropriate dose and duration of electrical stimulation can achieve various physiological responses [42]. Hee-Kyung et al. [14] showed that different combinations of frequency and electrical current intensity could produce an increase in sympathetic activity (low frequency [5 Hz] and electric stimulation level associated with muscle contraction or high frequency [100 Hz] and electrical stimulation level associated with tolerance level). Accordingly, Noble et al. [15] proposed that frequencies between 10 and 20 Hz and electrical current intensity associated with contractile activity also produce an increase in sympathetic activity. In contrast, Hee-Kyung et al. [14] obtained results similar to those of our study and showed a decrease in sympathetic activity associated with a high frequency [100 Hz] and with electrical stimulation level equivalent to sensitivity level. Second, differences between studies could be due to the measurement instrument used in the evaluation, since there are differences in the information provided by Doppler ultrasonography and a portable device such as First Beat^®^.

We also believe that the classroom environment and factors within it could have interfered with related changes in the physiological response such as prior experience among experienced and inexperienced students [43]. Therefore, we analysed participants’ attitudes and the learning environment in the classroom. Students from both groups scored an average of 23–24 points on the PPAS, which is much less than the threshold of 37.5 that is considered to indicate apprehension regarding the application of electrical current [23]. In addition, the classroom environment in the laboratory practice which was measured using LEQ obtained high scores (M = 116, SD = 26.54) in the dimensions of motivation and personal relationships [24]. Another factor that may influence autonomous responses is the degree of training in the procedures practiced in class. Clemente-Suárez et al. [11] analysed the influence of clinical simulation training on the autonomous response in psychology students. These authors concluded that with a higher degree of training (from three practices), the levels of sympathetic activity decreased significantly. Given this, the fact that our participating students had carried out at least five practices before we recruited the sample should be considered. However, these significant decreases in the indicators of sympathetic activity were only observed in the EG. In addition, we want to highlight that our students attended the electrotherapy laboratory practice session in the morning and were not sleep deprived, since this could be considered another factor related to academic performance [5].

### 4.1. Practical Implications

The analysis of HRV has been investigated due to its possible relationship with pain inhibition capacity [44] and has been applied to several pathological conditions [45,46]. However, investigations have been limited in students. The practical implications of these findings could be specified in the clinical information provided to the teacher and should be carefully addressed to avoid possible immediate adverse reactions (e.g., vasovagal reactions) [47]. Furthermore, the fact that it is the students who perceive in their own lumbar region the sensation of the passage of the current and its effects could lead to an improvement in the teaching received, and therefore to a better mastery of the procedure when it is to be applied in the clinical setting. 

### 4.2. Limitations

Firstly, the application of IFC has a local and segmental effect due to the stress-associated autonomic response caused. However, the local and segmental therapeutic mechanism of this technique is not clear. Therefore, future studies should clarify it. Secondly, this study analysed only healthy, adult male volunteers. There is a higher prevalence of parasympathetic activity in women that is associated with increased HRV [48]. Future studies should analyse the response of the IFC technique at the autonomic level in women. Thirdly, despite previous studies having been carried out in the university context [1,10], in this study the participants adopted a passive role (they received the passage of the current as patients) in the training session with the electric current. This distinctive feature makes the influence of these practical interventions on academic performance unclear. Finally, although the information transmitted to the sample subjects was controlled, the placebo effect in the electrotherapy group could be considered a potential limitation. Equally, the possible development of habituation or adverse reaction in sham group was controlled with some of the inclusion criteria of the study. Specifically, the fact that we measured personal psychological apprehension as a psychosocial covariate in both groups (Table 1) and that the participants had prior knowledge of the different sensory and contraction responses (perceptible and non-perceptible) of the electrophysical agents without having studied in detail the effects of interferential currents before the start of the study are factors that also control for this possible bias.

## 5. Conclusions

In conclusion, an electrotherapy training session with interferential current in male physiotherapy students who receive it produces an increase in parasympathetic activity and decrease in sympathetic activity (detected by HRV). It appears that the changes observed could be due to the IFC technique; however, future studies are needed to determine the effects in other educational settings.

## Figures and Tables

**Table 1:** Baseline clinical and demographic features of the sample.

	Total Sample (*n* = 90)	Electrotherapy Group (*n* = 46)	Sham Electrotherapy Group (*n* = 44)	*p* Value *
Mean age (years)	21 (4.67)	22 (5.91)	21 (2.80)	0.25
Height (cm)	178.58 (6.41)	179.33 (7.27)	177.80 (5.41)	0.26
Weight (kg)	73.86 (9.77)	75.23 (8.77)	72.42 (10.63)	0.18
Body Mass Index	23.15 (2.44)	23.51 (2.52)	22.77 (2.32)	0.15
PPAS	21.49 (8.07)	20.39 (7.51)	22.64 (8.56)	0.19
LEQ	116 (26.54)	115 (31.23)	119 (25.17)	0.23
rMSSD	45.12 (19.35)	42.78 (17.17)	47.57 (21.31)	0.24
pNN50	8.31 (2.98)	8.39 (3.26)	8.21 (2.68)	0.77
SD1	32.65 (13.00)	32.31 (14.49)	33.02 (11.38)	0.79
SD2	74.86 (27.13)	73.02 (23.45)	76.79 (30.67)	0.51
SS	15.38 (5.95)	15.34 (4.70)	15.42 (7.08)	0.95
RATIO S/PS	0.64 (0.63)	0.63 (0.45)	0.65 (0.78)	0.86

Data are reported as mean (SD). PPAS, Personal Psychological Apprehension Scale; Mean HR = Average heart rate, beats per minute (bpm); SD1 = transverse axis of Poincaré plot millisecond (ms); SD2 = longitudinal axis of Poincaré plot, SS = stress score (inverse of diameter SD2 × 1000); S/PS ratio = quotient of SS and SD1. * Statistically significant differences (*p* ≤ 0.05) between-groups statistical significance (one-factor ANOVA).

**Table 2:** Baseline, post-intervention, and mean score changes in the heart rate variability parameters and percentage changes for each of the variables in both groups.

Group	Baseline	Post-Intervention	Within-Group Mean Changes	Percentage Change (%)	*d*	Between-Groups Mean Changes (Percentage Total Differences)
rMSSD (ms)						
Sham electrotherapy Electrotherapy	47.57 (21.31) 42.78 (17.17)	53.55 (23.16) 50.69 (20.33)	5.97 [1.04 to −10.91] * 7.91 [3.90 to 11.91] **	12.5 18.5	0.27 0.42	2.86 [−6.25 to 11.98] (6%)
pNN50 (%)						
Sham electrotherapy Electrotherapy	8.21 (2.68) 8.39 (3.26)	9.91 (2.99) 10.91 (3.53)	1.70 [1.34 to 2.06] ** 2.52 [2.08 to 2.95] **	20.7 30	0.59 0.74	1.00 [−0.37 to 2.38] (9.3%)
SD1 (ms)						
Sham electrotherapy Electrotherapy	33.02 (11.38) 32.31 (14.49)	39.94 (15.40) 45.30 (13.76)	6.92 [3.07 to 10.77] * 12.99 [10.19 to 15.79] **	21 40	0.51 0.92	5.36 [−0.76 to 11.47] (19%)
SD2 (ms)						
Sham electrotherapy Electrotherapy	76.79 (30.67) 73.02 (23.45)	80.70 (25.10) 97.73 (30.49)	3.91 [1.96 to 9.78] 24.71 [16.29 to 33.13] **	5 34	- 0.91	17.03 [5.30 to 28.71] ^†^ (29%)
SS (ms)						
Sham electrotherapy Electrotherapy	15.42 (7.08) 15.34 (4.70)	16.53 (7.09) 12.60 (5.87)	1.11 [0.43 to 2.65] 2.74 [1.01 to 4.45] *	7 −18	- 0.51	3.85 [1.20 to 6.66] ^†^ (−25%)
RATIO S/PS						
Sham electrotherapy Electrotherapy	0.65 (0.78) 0.63 (0.45)	0.55 (0.40) 0.33 (0.20)	0.10 [−0.05 to 0.26] 0.30 [0.17 to 0.41] **	−15 −48	- 0.86	0.20 [0.07 to 0.34] ^†^ (−33%)

Interventions in the sham electrotherapy group (SEG) and electrotherapy group (EG) consisted in a IFC intervention without and with current, respectively. SD1 = transverse axis of Poincaré plot millisecond (ms); SD2 = longitudinal axis of Poincaré plot; SS = stress score (inverse of diameter SD2 × 1000); S/PS ratio = quotient of SS and SD1. Data are reported as mean (SD) or [95% confidence level]. * Indicates statistically significant within-group differences (*p* < 0.05). ** Indicates statistically significant within-group differences (*p* < 0.001). ^†^ Indicates statistically significant between-group differences (*p* < 0.05).

## Data Availability

Not applicable.
